# Cost of integrated immunization campaigns in Nigeria and Sierra Leone: bottom-up costing studies

**DOI:** 10.1186/s12913-024-11809-z

**Published:** 2024-11-01

**Authors:** Laura Boonstoppel, Flavia Moi, Christina Banks, Florence Sibeudu, Divine Obodoechi, Kyle Borces, Obinna Onwujekwe, Logan Brenzel

**Affiliations:** 1 ThinkWell, Geneva, Switzerland; 2 ThinkWell, Washington, D.C., USA; 3 Nnamdi Azikiwe University, Nnewi campus, Awka, Nnewi, Nigeria; 4 University of Nigeria, Enugu, Nigeria; 5 Bill & Melinda Gates Foundation, Seattle, USA

**Keywords:** Immunization, Campaign, Costing, Integration, Sierra Leone, Nigeria, Yellow fever vaccine, Meningitis A vaccine, Measles-rubella vaccine, Oral polio vaccine

## Abstract

**Background:**

To improve the efficient use of scarce resources, low- and middle-income countries and development partners are increasingly encouraged to deliver multiple vaccines and other interventions in a single integrated campaign. However, little is known regarding the cost of delivering vaccines through integrated campaigns, and the extent to which efficiencies are achieved. To fill this evidence gap, we estimated the cost of integrated immunization campaigns in Nigeria and Sierra Leone, and the potential savings from integration.

**Methods:**

We conducted a retrospective ingredients-based costing study from a payer perspective of a campaign held in 2019 in Sierra Leone with measles-rubella vaccine and oral polio vaccine, during which nutrition supplements were also offered in part of the country, and yellow fever campaigns held in three states in Nigeria in 2019 and 2020, where in one state (Anambra) meningococcal A vaccines were co-delivered. We collected data from 108 health facilities, all relevant administrative levels, and implementing partners. We estimated the full financial and economic cost of each campaign, the average unit cost of delivery, as well as the cost by activity and resource type. We also estimated the cost savings from integration in Anambra state by modelling out the cost of the alternative of two standalone campaigns.

**Results:**

The average financial delivery cost was $0.34 per dose in Sierra Leone, and the economic cost was $0.73 per dose. In Nigeria, the financial cost per dose was $0.29–$0.35 across the three states, and the economic cost per dose was $0.62–$0.85. Facilities and wards delivering more doses achieved a lower financial and economic unit cost of delivery, demonstrating evidence of economies of scale. We estimated that Anambra may have saved at least $1,204,133 in financial resources by integrating yellow fever and meningitis A vaccine delivery, amounting to $0.17 per dose delivered. When including opportunity costs, the economic cost saving was estimated at $0.34 per dose delivered.

**Conclusions:**

The study offers evidence on what it costs to deliver integrated campaigns, and shows that integrated delivery is likely to result in significant cost savings. Where high delivery volumes can be achieved, integrated campaigns can benefit from economies of scale. The findings can be used to inform planning and budgeting for immunization campaigns in low- and middle-income countries.

**Supplementary Information:**

The online version contains supplementary material available at 10.1186/s12913-024-11809-z.

## Introduction

Immunization campaigns have historically been a very effective tool to prevent or react to a disease outbreak, to rapidly achieve herd immunity when introducing a new vaccine in the routine program, and to target specific areas or population segments missed by the routine immunization program. Campaigns in low- and middle-income countries are often heavily reliant on donor support, and vertically funded and implemented, leading to a multitude of single-antigen campaigns. While financing for immunization programs is expected to decline over the coming decade, countries are facing an ambitious catch-up agenda to address immunization coverage declines caused by the Covid-19 pandemic [1, 2]. To improve the efficient use of scarce resources, countries and partners are increasingly encouraged to deliver multiple vaccines and other interventions in a single integrated campaign [3, 4]. 

Although countries are increasingly implementing integrated immunization campaigns, little is known regarding the cost of delivering vaccines through such campaigns, and the extent to which efficiencies are achieved. A systematic review of immunization delivery cost evidence published in 2019 found only 12 studies reported cost estimates on delivery through campaigns, all delivering only a single antigen [5]. Since then, one study has estimated the cost savings of measles and meningitis A vaccine delivery in three Nigerian states, though this covered only the incremental, financial delivery costs, and was not based on primary data from implementation level [6]. 

To fill the evidence gap on the cost of conducting immunization campaigns, and integrated campaigns in particular, we conducted an ingredients-based, bottom-up costing study of the cost of integrated immunization campaigns in two West African countries: a campaign in Sierra Leone with measles-rubella (MR) vaccine and oral polio vaccine (OPV), during which nutrition supplements were also offered in part of the country, and yellow fever (YF) campaigns in Nigeria, where in one of the three states in our study, meningococcal A (MenA) vaccines were co-delivered. We estimated the total costs of the campaigns, the unit cost of delivery, and modelled the potential financial and economic cost savings from integrated delivery compared to the alternative of standalone campaigns.

## Methods

### Study setting

In Sierra Leone, the campaign was conducted nationwide over 7 days in June 2019, with the primary aim of covering all children aged between 9 months and 15 years with MR, ahead of a switch from measles to MR in the routine program. In addition, OPV was administered to all children under 5, and in half of the country’s districts, Vitamin A and deworming supplements were delivered to children aged 6–59 months and 12–59 months respectively. The campaign was coordinated by the Ministry of Health and Sanitation, with support from the World Health Organization (WHO), United Nations Children’s Fund (UNICEF), and other partners. School-based delivery was used exclusively for MR, and other temporary fixed sites were set up at markets, transit points, community centers and other locations. Facilities also delivered the vaccines and supplements during the campaign, and mobile teams were deployed to move around in the communities as well. Most facilities also conducted mop-up activities to reach children who were not vaccinated during the campaign. The post-campaign coverage survey found 93% coverage for MR [7]. The other interventions were not included in the survey, and administrative coverage reported 120% for OPV, and 97% for each of the nutrition interventions [8]. 

In Nigeria, the campaigns included in this study were part of a 10-year yellow fever elimination plan. Under this plan, Nigeria has committed to vaccinating at least 80% of the target population (9 months to 44 years of age) by 2026, through a series of immunization campaigns, implemented in a phased manner. This study focused on the yellow fever campaigns conducted in 3 states: Katsina in September 2019, Rivers in February 2020, and Anambra in October 2020. In Anambra, the campaign was implemented alongside a MenA catch-up campaign targeting 1- to 6-year-olds. The campaigns were coordinated at federal level by the Department of Disease Control and Immunization of the National Primary Health Care Development Agency (NPHCDA), with support from the WHO, UNICEF, and other partners. In each state, local government areas (LGAs) took 10 days to complete the campaign. Campaign implementation was most often managed at ward level, and in some cases by facilities. Temporary posts were the most utilized delivery strategy, but facility-based delivery also took place, and a short mop-up was usually conducted as well. According to post-campaign coverage surveys, the campaigns achieved 83% coverage in Katsina, 82% in Rivers, and 76% (YF) and 96% (MenA) in Anambra [9]. 

The campaigns in Sierra Leone and Nigeria relied heavily on financing from Gavi, the Vaccine Alliance (Gavi), which fully funded the vaccines and a large share of the operational cost. This was complemented by funding from both governments. In Sierra Leone, the Global Polio Eradication Initiative also provided support for OPV, and the Canadian government supported the nutrition interventions. Where multiple vaccines and interventions were delivered, delivery was fully integrated, from campaign planning and coordination down to vaccine transport and service delivery.

### Study design

We followed an ingredients-based costing approach to capture all financial and economic costs of delivery from a payer perspective, which included the ministries of health and implementing partners. Costs incurred at the government body in charge of the immunization program at the national level, the sites that participated in the campaign, and all relevant administrative levels in between were included. The study covered activities conducted during the campaign, as well as planning, preparatory and wrap-up activities. All resource types were included, such as paid and volunteer labor, per diems, fuel costs, capital costs for vehicles and cold chain equipment, and the cost of vaccines, nutrition supplements, and related supplies. A full list of definitions of costed activities and resource types can be found in Supplemental Tables 1 and 2.

### Ethical considerations

The study was approved by the Health Research Ethics Committee from the University of Nigeria Teaching Hospital and by the Sierra Leone Ethics and Scientific Review Committee. All respondents signed informed consent forms ahead of being interviewed, and were assured that their personal details would be kept private, and excluded from the final dataset for the analysis.

### Sample

The three Nigerian states were purposively selected to represent the north, middle and south of the country. Within these, LGAs and wards were randomly selected. In Sierra Leone, 6 out of the country’s 14 districts were included, with half of these administering only vaccines during campaign, and the other half delivering both vaccines and nutrition interventions. The EPI selected two of the six districts: the riverain district of Bonthe, and the mountainous district of Koinadugu, as they were expected to have incurred higher delivery costs than other districts, and the other four districts were selected randomly. In Sierra Leone, stratified random sampling was used to select a mix of public and private facilities and hospitals in urban and rural areas, while in Nigeria, simple random sampling was applied to select the facilities. The Sample Design Optimizer [10] was utilized to explore efficient sampling designs that minimized the relative error and data collection cost. The final sample included 78 health facilities, and 30 in Sierra Leone, and data were also collected from the 10 relevant local government authorities in Nigeria, and 6 district health offices in Sierra Leone, as well as relevant state and national level offices, and implementing partners.

### Data collection & analysis

Data were collected retrospectively between October 2019 and May 2021 using standardized Excel-based questionnaires, tailored to the different administrative levels. Unit prices were obtained at local level, and where not available, national or global level estimates were used instead. This was the case for salary data, fuel prices, replacement prices and useful life measurements, building costs, vaccine prices, and international shipment costs. For other data that were missing at local level, such as standard facility opening hours, incinerator running costs and the number of syringes used, we filled gaps based on data from similar sites and settings. The cost of volunteer time was estimated at the equivalent of the salary grade of the cadre that best represented their responsibilities, or at minimum wage level in the case of day laborers.

Shared costs were allocated to the campaign and to activities based on time spent (for labor and vehicle costs), or the proportion of space used in the cold chain (for cold chain-related costs). Capital costs were annualized over their useful life, using replacement prices, and straight-line depreciation. For the economic costs, they were discounted using a 3% rate [11]. Costs were standardized to 2020 currency using the International Monetary Fund (IMF) consumer price index, and subsequently converted into United States dollars (USD) using the World Bank official exchange rates [12, 13]. 

The cost per dose delivered was calculated for each administrative level, weighted by the volume delivered and inverse probability of sampling [14, 15]. The total delivery cost per dose was obtained by aggregating the unit cost at each level, and the total cost of the campaigns was calculated by multiplying the unit cost by the total number of doses delivered. In the case of Sierra Leone, a national level average was estimated, as well as group estimates for the districts that delivered nutritional supplements, and the group of districts that did not. In the case of Nigeria, we estimated average cost levels for each of the three states. As the campaigns in the three states took place during different time periods, and the national level strategy encompasses a 10-year period, we did not extrapolate the state level findings to a national level average. The datasets from Sierra Leone and Nigeria can be found on Harvard Dataverse [16, 17]. Two-sample t-tests were conducted to test for statistical significance between the various groups (*p* < 0.05). A bootstrap regression using 1000 replications was also run to generate the confidence intervals (CI).

To estimate the cost savings from integration in Nigeria, we modelled the cost of two standalone campaigns with YF and MenA in Anambra, and compared these with the cost estimated by our bottom-up costing study of the integrated YF/MenA campaign in Anambra. The cost of a YF standalone campaign was estimated based on the practices in Katsina and Rivers, and for the standalone MenA campaign, we leveraged assumptions on MenA campaign practices from other sources [18]. In instances of uncertainty around assumptions, we opted for the scenario that would yield the lowest cost for a standalone campaign. This approach ensured that the findings of our analysis reflect a conservative minimum of the potential savings that could be achieved through integration. The assumptions for the scenarios can be found in Supplemental Table 3. To exclude the impact of inflation from the model, we assumed that the two standalone campaigns would take place in the same year.

## Results

### The total cost of the campaigns

The total financial cost of delivering the campaigns (excluding the cost of the vaccines) was estimated at between $1,804,205 and $2,578,029 across the sampled states in Nigeria and $1,713,549 in Sierra Leone (Table 1). The total economic delivery cost (which includes the value of labor and other opportunity costs) was estimated to range from $4,148,610 to $5,328,639 in Nigeria and $3,641,515 in Sierra Leone. The total cost of vaccines and other interventions delivered during the campaigns was estimated at $2,376,604 in Sierra Leone and between $8,590,299 and $9,548,766 across Nigerian states.

**Table 1:** The total delivery cost of the campaigns by state and country in 2022 USD

Country / state	Vaccines and nutrition supplements	Financial delivery cost	Economic delivery cost
Sierra Leone	$ 2,376,604	$ 1,713,549	$ 3,641,515
Katsina (Nigeria)	$ 9,548,766	$ 2,290,290	$ 4,148,610
Rivers (Nigeria)	$ 8,590,299	$ 1,804,205	$ 5,328,639
Anambra (Nigeria)	$ 8,642,407	$ 2,578,029	$ 5,157,628

### Delivery cost per dose

Table 2 shows the results per dose delivered. The average financial cost of delivering a dose of MR, OPV or a nutritional supplement during the campaign in Sierra Leone was $0.34, and the economic cost per dose was $0.73. In Nigeria, the financial cost of delivering a dose of YF varied from $0.29 in Rivers to $0.34 in Katsina, and the cost of delivering either a YF or MenA dose was $0.35 in Anambra. The economic delivery cost per dose varied slightly more from $0.62 to $0.85 across the three states. Including the cost of the vaccines and nutritional supplements, as well as the cost of freight and wastage, the total economic cost per dose of the campaign was $1.22 in Sierra Leone and up to $2.22 in Nigeria.

**Table 2:** Unit costs and delivery volume across geographies (weighted averages and 95% CI)

Geography	Interventions delivered	Total delivery volume (all interventions)	Median doses delivered per facility or ward (all interventions)	Financial delivery cost per dose	Economic delivery cost per dose	Vaccine/ supplement cost per dose*	Total cost per dose
Sierra Leone	MR, OPV, and nutrition supplements in half of the districts	5,060,899	3,964	$0.34 [$0.27-$0.42]	$0.73 [$0.57-$0.89]	$0.49**	$1.22 [$1.06–$1.38]
Katsina (Nigeria)	YF only	6,695,692	3,013	$0.34 [$0.19–$0.49]	$0.62 [$0.47–$0.78]	$1.43 [$1.19–$1.66]	$2.05 [$1.66–$2.44]
Rivers (Nigeria)	YF only	6,279,531	17,186	$0.29 [$0.25–$0.32]	$0.85 [$0.68–$1.02]	$1.37 [$1.25–$1.48]	$2.22 [$1.93–$2.50]
Anambra (Nigeria)	YF and MenA	7,293,668	12,650	$0.35 [$0.22–$0.49]	$0.71 [$0.53–$0.89]	$1.18 [$1.13–$1.24]	$1.89 [$1.65–$2.13]

* Includes freight and wastage. For Sierra Leone and Anambra, this represents the weighted average of all vaccines and supplements used. ** No CI as based on national level volumes rather than facility level data

### Delivery cost drivers

The financial delivery cost of the campaigns was mostly driven by per diem and allowances, vaccine injection and safety supplies, and transport and fuel costs (Table 3). The economic delivery costs were primarily driven by the cost of labor. Unpaid labor includes the value of volunteers, and the significant number of health workers that were not on payroll, and only received per diems or a small stipend. In Sierra Leone, 70% of the health workers involved in the campaign were not on payroll, and in Nigeria, this varied from 56% in Katsina to 95% in Anambra. The Anambra campaign took place during the COVID-19 pandemic, which explains why the use and cost of personal protective equipment (PPE), and infection prevention and control (IPC) materials such as hand sanitizer, soap, and buckets, was greater here. A breakdown of costs by activity can be found in Supplemental Table 4.

**Table 3:** Cost of delivering a dose by type of resource (% share of the total unit cost)

Country/state	Sierra Leone		Anambra state, Nigeria		Katsina state, Nigeria		Rivers state, Nigeria	
Type of cost	Financial	Economic	Financial	Economic	Financial	Economic	Financial	Economic
**Labor costs**	**$0.02** **(5%)**	**$0.39** **(53%)**	**$0.01** **(1%)**	**$0.34** **(49%)**	**$0.001** **(< 1%)**	**$0.27** **(43%)**	**$0.002** **(1%)**	**$0.56** **(66%)**
Paid labor	$0.02 (5%)	$0.20 (28%)	$0.01 (1%)	$0.16 (22%)	$0.001 (< 1%)	$0.19 (30%)	$0.002 (1%)	$0.31 (37%)
Volunteer labor	-	$0.19 (26%)	-	$0.19 (27%)	-	$0.08 (13%)	-	$0.25 (29%)
**Other operating costs**	**$0.32** **(94%)**	**$0.33** **(44%)**	**$0.35** **(99%)**	**$0.35** **(50%)**	**$0.34** **(100%)**	**$0.34** **(55%)**	**$0.29** **(99%)**	**$0.29** **(34%)**
Per diems, transport allowances, community incentives	$0.14 (40%)	$0.14 (19%)	$0.09 (26%)	$0.09 (13%)	$0.09 (26%)	$0.09 (15%)	$0.09 (33%)	$0.09 (11%)
Transport and fuel	$0.06 (16%)	$0.06 (8%)	$0.09 (27%)	$0.09 (13%)	$0.10 (29%)	$0.10 (16%)	$0.04 (15%)	$0.04 (5%)
Vaccine injection and safety supplies	$0.06 (18%)	$0.06 (9%)	$0.08 (23%)	$0.08 (11%)	$0.09 (28%)	$0.09 (15%)	$0.09 (31%)	$0.09 (11%)
PPE & IPC	-	-	$0.02 (5%)	$0.02 (3%)	$0.005 (1%)	$0.005 (1%)	$0.002 (1%)	$0.002 (< 1%)
Workshops and meetings	$0.02 (5%)	$0.02 (2%)	$0.03 (8%)	$0.03 (4%)	$0.02 (7%)	$0.02 (4%)	$0.01 (5%)	$0.01 (2%)
IEC and other printing costs, stationery and other supplies, communication	$0.02 (7%)	$0.02 (3%)	$0.03 (8%)	$0.03 (4%)	$0.02 (7%)	$0.02 (4%)	$0.04 (13%)	$0.04 (5%)
Vehicle maintenance, incinerator running costs	$0.02 (6%)	$0.02 (3%)	$0.01 (2%)	$0.01 (1%)	$0.004 (1%)	$0.004 (1%)	$0.002 (1%)	$0.002 (< 1%)
Cold chain repairs, energy costs	$0.002 (1%)	$0.002 (< 1%)	$0.00004 (< 1%)	$0.002 (< 1%)	$0.0001 (< 1%)	$0.003 (< 1%)	$0.00002 (< 1%)	$0.001 (< 1%)
**Capital costs** (Cold chain equipment, incinerators, vehicles, other)	**$0.002** **(< 1%)**	**$0.02** **(2%)**	**$0.00003** **(< 1%)**	**$0.01** **(2%)**	**$0.00003** **(< 1%)**	**$0.01** **(2%)**	**$0.00003** **(< 1%)**	**$0.002** **(< 1%)**

### Subnational and group differences

Table 4 shows the unit cost of delivery by sampled district (for Sierra Leone) and LGA (for Nigeria). The two districts in Sierra Leone that were selected due to their geographical challenges (Koinadugu and Bonthe) were among those with the highest cost per dose levels. Districts and LGAs where facilities and wards delivered more vaccine doses and nutritional supplements tended to have a lower unit cost of delivery (both financial and economic). Many of the districts in Sierra Leone that delivered nutritional interventions in addition to the vaccines achieved a relatively low unit cost of delivery, demonstrating economies of scale from co-delivery. On the other hand, although Anambra was the only state in the study that offered MenA vaccines alongside YF during the campaign, wards in Anambra often delivered fewer doses than wards in other states where only YF was delivered, and thus no economies of scale were observed. In Sierra Leone, the cost difference between the group of vaccination sites that delivered only MR and OPV, compared with the group that delivered nutritional supplements as well, was not statistically significant.

**Table 4:** Unit costs and delivery volume across geographies (weighted averages and 95% CI)

Geography (country/state/ward)	Interventions delivered	Total delivery volume (all interventions)	Median doses delivered per facility/ward (all interventions)	Financial delivery cost per dose	Economic delivery cost per dose
**Sierra Leone**	**MR, OPV, and nutrition**	**1,044,979**	**4,286**	**$0.30 [$0.39-$0.21]**	**$0.66** **[$0.51-$0.82]**
Bombali	MR, OPV, and nutrition	460,784	6,543	$0.21	$0.55
Bonthe	MR, OPV, and nutrition	149,341	3,381	$0.39	$0.74
Port Loko	MR, OPV, and nutrition	434,854	8,497	$0.39	$0.74
**Sierra Leone**	**Only MR + OPV**	**763**,**856**	**3**,**366**	**$0.42** **[$0.31-$0.52]**	**$0.85** **[$0.54-$1.17]**
Koinadugu	Only MR + OPV	238,592	3,450	$0.53	$1.28
Pujehun	Only MR + OPV	213,048	4,142	$0.43	$0.93
Tonkolili	Only MR + OPV	312,216	2,549	$0.35	$0.61
**Katsina (Nigeria)**	**YF only**	**6**,**695**,**692**	**3**,**013**	**$0.34** **[$0.19–$0.49]**	**$0.62** **[$0.47–$0.78]**
Funtua	YF only	323,333	2,605	$0.25	$0.50
Ingawa	YF only	178,976	3,080	$0.48	$0.81
**Rivers (Nigeria)**	**YF only**	**6**,**279**,**531**	**17**,**186**	**$0.29** **[$0.25–$0.32]**	**$0.85** **[$0.68–$1.02]**
Eleme	YF only	254,806	28,600	$0.23	$0.81
Etche	YF only	305,312	9,282	$0.21	$0.63
Obio-Akpor	YF only	770,806	18,386	$0.30	$0.99
Oyibo	YF only	156,069	17,186	$0.42	$1.18
**Anambra (Nigeria)**	**YF + MenA**	**7**,**293**,**668**	**12**,**650**	**$0.35** **[$0.22–$0.49]**	**$0.71** **[$0.53–$0.89]**
Awka North	YF + MenA	194,992	14,794	$0.57	$1.55
Njikoka	YF + MenA	238,545	11,161	$0.43	$0.83
Orumba North	YF + MenA	191,357	5,150	$0.20	$0.52
Orumba South	YF + MenA	330,531	18,764	$0.32	$0.57

### Estimated cost savings from integration

Table 5 shows the findings from the cost estimation of two hypothetical standalone campaigns, in comparison with the cost of the integrated campaign that took place in Anambra state, Nigeria. The cost of two standalone campaigns in Anambra, one to deliver YF vaccines and one to deliver MenA vaccines to their respective target populations, was estimated at a combined financial cost of approximately $3,782,162. When including opportunity costs, the total economic cost of these two campaigns would have been $7,612,273. Compared with the delivery cost of the integrated YF/MenA campaign that Anambra conducted, we estimated that Nigeria saved $1,204,133 in financial expenditures by integrating the delivery of the two antigens, and a total of $2,454,645 in terms of economic cost. Per vaccine dose delivered, the financial cost savings were estimated at $0.17, and $0.34 when including opportunity costs. The financial cost savings were mainly driven by a reduction in per diems and allowances, while the savings in opportunity costs were related to the reduced time spent by staff at all levels of the health system.

**Table 5:** Estimated minimum cost of two standalone campaigns in Anambra compared with the cost of the integrated campaign

Cost scenarios for Anambra state	Total financial delivery cost	Total economic delivery cost
Integrated campaign (YF and MenA)	$ 2,578,029	$ 5,157,628
Estimated minimum cost of two standalone YF and MenA campaigns	$ 3,782,162	$ 7,612,273
Estimated savings from campaign integration	$ 1,204,133	$ 2,454,645
Savings per dose delivered	$ 0.17	$ 0.34

## Discussion

This study found that the financial cost of delivering MR vaccine, OPV, and nutritional supplements through a nationwide campaign in Sierra Leone was on average $0.34 per dose delivered, and the economic cost per dose was $0.73. The financial cost of the YF-only and YF/MenA campaigns in Nigeria was on average $0.29 to $0.35 per dose, and the economic cost per dose was $0.62-$0.85. The largest drivers of the financial delivery cost of both campaigns were per diem and allowances, vaccine injection and other supplies, and transport and fuel costs, likely because in both countries, most of the vaccine doses and nutritional supplements were delivered through temporary sites. The Anambra campaign took place during the COVID-19 pandemic, which explains why the use and cost of PPE, and IPC materials such as hand sanitizer, soap, and buckets, was greater here. The economic costs were largely driven by the value of paid and volunteer labor.

Our findings on the cost of the MR/OPV campaign in Sierra Leone are a little high compared with the existing literature on campaigns with a measles containing vaccine. A study of an MR catch-up campaign in India found a financial unit cost of delivery of $0.16-$0.34 (2019 USD) [19] and a study of a measles campaign in Benin found $0.90 (2011 USD) including the cost of the vaccine [20]. A 2011 study in Cameroon found a financial delivery cost of only $0.14 per dose, though this only included district and intermediate level costs [21]. Our higher cost per dose is likely because measles campaigns do not normally implement house-to-house delivery, and unfortunately we could not find published cost evidence from OPV house-to-house campaigns to compare our results with.

Our findings on the cost of the YF and YF/MenA campaigns in Nigeria are low compared to the only other published study on the cost of YF campaigns of a campaign conducted in Abidjan in 2009, which found a financial cost of $0.43 per dose (in 2020 USD) [22], though higher than found in other studies of MenA campaigns ($0.42 in Burkina Faso and $0.22 in Chad). The latter campaigns likely had a lower cost per dose as they targeted a much larger cohort than the mini catch-up in Anambra [23, 24]. The economic cost of delivering yellow fever and meningitis A in Nigeria was also lower than the findings of a study of measles SIAs in Anambra ($0.81), especially when considering that this estimate excluded costs incurred above LGA level, vaccine supplies and waste management costs [25]. 

Both campaigns in our study were heavily reliant on support from Gavi, the Vaccine Alliance. Gavi’s operational cost support for campaigns amounts to $0.45-$0.65 per targeted person, depending on the country’s transition phase. While most districts and wards in Sierra Leone and Nigeria in our study had a lower financial cost of delivery than the Gavi subsidy, some did exceed it. Therefore, although the average level of Gavi support is likely sufficient to support immunization campaigns, the wide variation in delivery cost observed signals a need for tailoring support to subnational level needs.

We observed that facilities and wards delivering more doses achieved a lower financial and economic unit cost of delivery, demonstrating evidence of economies of scale. While high delivery volumes could be achieved through co-delivery of multiple antigens and interventions (as was the case in Sierra Leone), we also found that co-delivery alone fell short of realizing this goal (as observed in Anambra). Nevertheless, even in the absence of economies of scale, cost savings from integrations could be significant. Our analysis showed that Anambra may have saved at least $1,204,133 by integrating YF and MenA delivery, amounting to $0.17 per dose delivered. This is similar to the savings of $0.20 (2019 USD) estimated by a study of integrated measles/MenA and standalone measles campaigns in Nigeria [6]. Including the value of existing resources used in implementing a campaign, we estimated the economic cost saving from integration at $0.34 per dose. This demonstrates that savings from campaign integration benefit both donors that fund immunization campaigns, as well as governments, by reducing a significant burden on the existing immunization program’s resources.

The cost evidence presented in our study carries several limitations. There is a risk of recall bias in both studies due to the length of time which had elapsed between the campaign and data collection, as data collection took place between 1 and 19 months after each campaign. The assumptions and interpolations used to fill data gaps resulted in an additional margin of error around our cost estimates, though we expect this to be small, as this mainly related to cost components for which variability is limited (such as the number of syringes per vaccine dose) or components that were not cost drivers (such as incinerator running costs). Missing data on the catchment population size, target population size, and weak subnational level coverage data, meant that cost estimates could not be analyzed against output indicators.

## Conclusion

This study offers concrete cost evidence on integrated campaigns in Sierra Leone and Nigeria, which can be used for planning and budgeting in low- and middle-income country settings. Our findings show that integrated delivery of multiple antigens and strategic inclusion of other interventions is likely to result in significant cost savings, and when high delivery volumes can be achieved, integrated campaigns might benefit from economies of scale. These findings can be used to inform donor funding strategies for immunization campaigns in low- and middle-income countries, to improve the efficiency and effectiveness of the delivery of immunization and other essential health services through various delivery strategies, and ultimately improve health outcomes.

## Supplementary Information

Supplementary Material 1.

## Data Availability

The datasets generated and analyzed during the current study are available in Harvard's Dataverse repository: - Nigeria: 10.7910/DVN/KOJ2LY - Sierra Leone: 10.7910/DVN/NEEBLN

## Abbreviations

CI: Confidence interval
Gavi: Gavi, the Vaccine Alliance
IMF: International Monetary Fund
IPC: Infection prevention and control
LGA: Local government area
MenA: Meningitis A
MR: Measles-rubella
NPHCDA: National Primary Health Care Development Agency
OPV: Oral polio vaccine
PPE: Personal protective equipment
USD: Unites States dollar
WHO: World Health Organization
YF: Yellow fever
