# Supplementary material for: Cost of integrated immunization campaigns in Nigeria and Sierra Leone: bottom-up costing studies
Source: BMC Health Serv Res. 2024 Nov 1;24:1334. doi: 10.1186/s12913-024-11809-z (PMC11529071; doi:10.1186/s12913-024-11809-z)
Supplement: Supplementary file 1 — Supplementary Material 1. [file 12913_2024_11809_MOESM1_ESM.docx]

Supplemental table 1: Descriptions of cost activities

| **Campaign activity** | **Definition** |
| --- | --- |
| Campaign management | Time and resources spent on planning, budgeting, and managing the immunization program at various levels, including attendance at immunization-related meetings. General management of the health system has not been allocated here. |
| Vaccine collection, distribution and storage | Time and resources spent collecting vaccines and other campaign commodities at the airport or other distribution points, storing vaccines in national or subnational cold stores, and distributing vaccines down to the facilities, and to the temporary campaign sites. |
| Cold chain maintenance | Time and resources spent on cold chain maintenance at all levels. |
| Training | Time and resources spent attending and/or providing campaign-related training. All trainings held in the lead-up to the campaign have been considered fully campaign-specific. Training costs include the cost of venue, per diem for participants, cost of trainers, and reproduction of training materials. |
| Social mobilization and advocacy | Time and resources spent on mobilizing the community and households, and advocating for vaccination. This includes the costs of holding community meetings, printing flyers and educational materials, conducting events, and the cost of television and radio time etc. |
| Supervision | Time and resources spent on supervising subordinate or peer health or community workers, including staff time and transport costs etc. |
| Service delivery: facility-based | Time and resources spent on the act of administering the interventions to the target groups within the facility/compound. |
| Service delivery: temporary posts | Time and resources spent on traveling to and from temporary posts and the act of administering the interventions to the target groups at these sites. Temporary posts could include schools, market places, and churches. |
| Service delivery: mobile teams/outreach (Sierra Leone only) | Time and resources spent traveling to and from mobile sites and the act of administering the interventions at these sites. |
| Service delivery: sweeping/mop-up | Time and resources spent on traveling to and from sites and the act of administering the interventions to the target population not reached during the campaign. |
| Waste management | Time and resources spent on disposing sharps and infectious non-sharp waste. |
| AEFI management | Time and resources spent following-up on adverse events following immunization (AEFI). |
| Record-keeping, HMIS, monitoring and evaluation | Time and resources spent on health management information systems (HMIS), data entry and analysis, including maintaining stock registers and records of children vaccinated, completing reports and analysing, monitoring, and evaluating campaign data. |

Supplemental table 2: Descriptions of resource types

| **Resource type** | **Description** |
| --- | --- |
| **Operating costs** | |
| Paid labor | Allocation of salaried labor to campaign-related activities. Salaries are fully loaded thus including any regular fringe benefits. Includes regular monthly stipends paid to volunteer workers. |
| Volunteer labor | Estimation of the market value of volunteer labor used for campaign-related activities. For unpaid health workers, an equivalent salary grade was collected. |
| Workshops and meetings | Costs related to workshops, trainings and meetings, including the venue and refreshments provided on the day, but not including transport or per diem costs. |
| Per diem and travel allowances | Any allowances paid to campaign staff and health workers for campaign-related activities. |
| Transport and fuel | Cost of bus fares, boat travel/hire, vehicle hire, and the cost of fuel for campaign-related transport. |
| Vaccine injection and safety supplies | Cost of auto-disabled syringes, reconstituting syringes, safety boxes, personal protective equipment and other supplies used for the administration of vaccines during the campaign. |
| Stationery and other supplies | Cost of stationery and other supplies used for the campaign. |
| IEC and other printing costs | The cost of printing immunization cards, training materials, radio jingles, tv ads and other information, education, and communication (IEC) materials that are campaign-related. |
| Communication | Costs related to purchasing airtime and mobile data for the purpose of the campaign, as well as a portion of regular phone and internet connection charges. |
| Other recurrent | Any other recurrent costs incurred during the campaign, this includes the cost of running incinerators used for the campaign and equipment rental costs. |
| Vehicle maintenance | Cost of maintaining vehicles (of all types) used for campaign-related activities. |
| Cold chain repairs and energy costs | The cost of repairing existing cold chain equipment and running the cold chain (electricity etc.). |
| Utilities | Costs related to building overheads, including maintenance, and utilities with a portion of these costs allocated to the campaign. These costs were included for Sierra Leone but not for Nigeria. |
| **Capital costs** | |
| Buildings | Value of the building space used to deliver and store vaccines. These costs were included for Sierra Leone but not for Nigeria. |
| Cold chain equipment | Value of all cold chain equipment used to store and transport vaccines. |
| Vehicles | Value of all vehicles and modes of transport used for the campaign. |
| Incinerators | Equipment used for incinerating waste at all levels. |
| Other equipment | Value of other equipment, such as generators, computers, printers, peripherals, phones, other medical equipment used for campaign-related activities. |

Supplemental table 3: Cost assumptions for standalone campaign scenarios in Anambra

| **Cost component** | **YF-only campaign scenario** | **MenA-only campaign scenario** |
| --- | --- | --- |
| Campaign duration | 10 days (which is the duration of both the standalone YF campaigns in Katsina and Rivers, and the integrated YF-MenA campaign in Anambra) | 5 days (duration of MenA mini catch-ups held in other states) |
| Service delivery, vaccine collection, distribution and storage, cold chain maintenance, waste management, AEFI management | Adjusted labor and other resources spent down to account for the lower delivery volume of a standalone YF campaign | Adjusted down to account for lower delivery volume of a standalone MenA campaign, as well as the shorter campaign duration |
| Delivery strategy mix | Assumed similar proportions of delivery strategies used (about 70% of doses delivered through temporary posts) | Assumed similar proportions of delivery strategies used (about 70% of doses delivered through temporary posts) |
| Vaccination team size | Reduced in line with the observed team size of YF standalone campaigns in Katsina and Rivers | Reduced in line with the observed team size of YF standalone campaigns in Katsina and Rivers |
| Campaign management meetings, social mobilization events & trainings | Same as the integrated campaign (as was the case for YF standalone campaigns) | Same as the integrated campaign (as was the case for YF standalone campaigns) |
| Supervision, per diem and travel allowances | 100% of the cost of the integrated campaign (as was the case for YF standalone campaigns) | Reduced to account for the shorter campaign duration |
| Record-keeping and reporting | Fewer recordkeepers per team, as was the case for YF standalone campaigns, and printing and stationery costs reduced to account for lower delivery volume | Fewer recordkeepers per team, as was the case for YF standalone campaigns, and printing and stationery costs reduced to account for lower delivery volume and shorter campaign duration |

Supplemental table 4: Cost breakdown by cost activity (cost per dose delivered in 2022 USD)

| **Country/state** | **Sierra Leone** | | **Anambra state, Nigeria** | | **Katsina state, Nigeria** | | **Rivers state, Nigeria** | |
| --- | --- | --- | --- | --- | --- | --- | --- | --- |
| **Type of cost** | **Financial** | **Economic** | **Financial** | **Economic** | **Financial** | **Economic** | **Financial** | **Economic** |
| Campaign management | $ 0.01 | $ 0.05 | $ 0.03 | $ 0.08 | $ 0.04 | $ 0.09 | $ 0.02 | $ 0.04 |
| Vaccine and commodity collection, distribution and storage | $ 0.01 | $ 0.03 | $ 0.02 | $ 0.04 | $ 0.02 | $ 0.04 | $ 0.02 | $ 0.03 |
| Cold chain maintenance | $ 0.001 | $ 0.01 | $ 0.0002 | $ 0.004 | $ 0.002 | $ 0.01 | $ 0.0004 | $ 0.003 |
| Training | $ 0.05 | $ 0.10 | $ 0.01 | $ 0.04 | $ 0.02 | $ 0.04 | $ 0.01 | $ 0.05 |
| Social mobilization | $ 0.03 | $ 0.11 | $ 0.07 | $ 0.12 | $ 0.05 | $ 0.10 | $ 0.07 | $ 0.19 |
| Supervision | $ 0.05 | $ 0.08 | $ 0.02 | $ 0.04 | $ 0.02 | $ 0.05 | $ 0.02 | $ 0.12 |
| Service delivery: facility-based | $ 0.04 | $ 0.06 | $ 0.03 | $ 0.05 | $ 0.06 | $ 0.07 | $ 0.02 | $ 0.06 |
| Service delivery: temporary sites | $ 0.06 | $ 0.10 | $ 0.14 | $ 0.21 | $ 0.10 | $ 0.11 | $ 0.10 | $ 0.17 |
| Service delivery: mobile teams/outreach | $ 0.06 | $ 0.08 | N/A | N/A | N/A | N/A | N/A | N/A |
| Service delivery: sweeping/mop-up | $ 0.01 | $ 0.03 | $ 0.002 | $ 0.004 | $ 0.01 | $ 0.01 | $ 0.004 | $ 0.02 |
| Waste management | $ 0.003 | $ 0.01 | $ 0.01 | $ 0.01 | $ 0.01 | $ 0.02 | $ 0.01 | $ 0.01 |
| AEFI management | $ 0.002 | $ 0.003 | $ 0.001 | $ 0.002 | $ 0.002 | $ 0.01 | $ 0.001 | $ 0.01 |
| Record-keeping, HMIS, monitoring and evaluation | $ 0.02 | $ 0.06 | $ 0.03 | $ 0.08 | $ 0.01 | $ 0.06 | $ 0.03 | $ 0.13 |
